# Cytoplasmic and nuclear events controlling Tax-mediated activation of the NF-κB pathway: involvement of TAB2, IKKgamma/NEMO and calreticulin

**DOI:** 10.1186/1742-4690-8-S1-A138

**Published:** 2011-06-06

**Authors:** Françoise Bex, Francesca Avesani, Maria G Romanelli, Julie Lodewick, Marco Turci, Carla Sampaio, Pierre Roger, Umberto Bertazzoni

**Affiliations:** 1 Institute for Microbiological Research J-M Wiame, University of Bruxelles, Bruxelles, B-1070, Belgium; 2 Department of Life and reproduction Sciences, Section of Biology and Genetics, University of Verona,Verona, Italy; 3 Institute of interdisciplinary Research, Université Libre de Bruxelles, Bruxelles, B-1070, Belgium

## 

The Tax oncoprotein of HTLV-1 initiates T-cell transformation by dysregulating cell cycle progression and inhibiting DNA damage responses. The subsequent genomic instability might result in constitutive activation of the NF-κB pathway observed in HTLV-1-transformed T lymphocytes. Our previous results indicated that differential modifications of Tax by ubiquitination or sumoylation controlled its retention either in the cytoplasm or in the nucleus, respectively. Here we show that Tax is targeted to pre-existing punctate cytoplasmic structures which contain the TNF-receptor associated protein 2 (TAB2). Colocalization of Tax with TAB2 in these cytoplasmic structures induced the recruitment of additional components involved in the NF-κB activation cascade, including the regulatory subunit of the IKK complex, IKKgamma/NEMO, the RelA subunit of NF-κB and TAX1-BP1, which are Tax interacting partners. Overexpression of TAB2 strongly stimulated Tax-mediated activation of the NF-κB pathway indicating that the concentration of these NF-κB factors in Tax-containing cytoplasmic punctate structures is a step important for activation of the NF-κB pathway by Tax. Interestingly, calreticulin, a multifunctional calcium buffering chaperone previously involved in the nucleocytoplasmic transport of nuclear receptors such as glucocorticoid and thyroid receptors, was also recruited by Tax in these cytoplasmic structures. The fact that overexpressed IKKgamma concentrated in nuclear foci that included calreticulin and that co-expression of Tax and IKKgamma led to the redistribution of both IKKgamma and calreticulin from the nucleus to the cytoplasm suggested that calreticulin might be involved in the nuclear export of these factors and their recruitment in TAB2-containing cytoplasmic punctate structures. The possibility that Tax activates the NF-κB pathway via initiation of events in the nucleus leading to the transport of IKKgamma from the nucleus to the cytoplasm will be discussed.

